# Solid Pseudopapillary Neoplasm of the Pancreas: A Clinicopathological Study of 12 Cases With Emphasis on Diagnostic Pitfalls

**DOI:** 10.7759/cureus.49858

**Published:** 2023-12-03

**Authors:** Abdelrazak Meliti, Jaudah Al-Maghrabi

**Affiliations:** 1 Pathology and Laboratory Medicine, King Faisal Specialist Hospital and Research Centre, Jeddah, SAU; 2 Pathology, Alfaisal University College of Medicine, Riyadh, SAU; 3 Department of Pathology, Faculty of Medicine, King Abdulaziz University, Jeddah, SAU

**Keywords:** solid pseudopapillary neoplasm, low malignant potential, neuroendocrine tumor, solid cystic papillary tumor, neoplasm of the pancreas

## Abstract

Introduction: Solid pseudopapillary neoplasm of the pancreas (SPNP) is a rare primary neoplasm with distinct clinicopathological features. The tumor most commonly occurs in younger (premenopausal) women and is typified by low malignant potential and an excellent overall prognosis.

Methods: A retrospective search over 20 years at two referral tertiary care institutions (King Faisal Hospital and Research Center and King Abdulaziz University Hospital, Jeddah, Kingdom of Saudi Arabia) revealed 12 female patients diagnosed with SPNPs. The reslts of ancillary studies performed at the time of diagnosis were also reviewed and placed in the context of current recommendations.

Results: The clinical and pathological findings were reviewed. All patients were females, aged 18 to 30 years. Eight patients presented with abdominal pain, of which two experienced significant weight loss, and four presented with abdominal mass/discomfort. The tumor size ranged from 1.5 and 15 cm. Two cases were initially diagnosed as neuroendocrine tumors (NETs). One of the cases presented as a multifocal disease. All patients were treated surgically with a follow-up period between one and 11 years. Only one patient presented with peritoneal metastasis after seven years of follow-up, but generally, all are doing well.

Conclusions: We have analyzed 12 SPNP cases in our population over 20 years (2001-2021) in this study. In brief, SPNP is a low-grade malignant potential tumor. Even though SPNP is a recognized entity, diagnostic challenges can arise particularly in the setting of limited sampling. Pathologists must be aware of the classic morphological features of SPNP and the characteristic profile of immunohistochemistry and be able to differentiate SPNP from other mimickers, especially well-differentiated NETs of the pancreas, and ultimately to avoid misdiagnosis and unnecessary oncologic treatment. Adequate surgical resection with negative margins is associated with an excellent outcome.

## Introduction

The solid pseudopapillary neoplasm of the pancreas (SPNP) is a distinct and relatively uncommon clinicopathological entity that Virginia K. Frantz first described in 1959 [1]. SPNP accounts for about 1-3% of all pancreatic tumors and usually occurs in young women, usually present in the third to fourth decades of life (mean age 35 years) [2]. The diagnosis of SPNP in the contemporary era is significantly improved owing to a better awareness of this entity among pathologists and improved diagnostic capacity from the imaging standpoint [3]. Nevertheless, pathologists must exercise extreme caution in the proper clinical context not to entertain the diagnosis of a neuroendocrine tumor (NET) until an accurate morphological evaluation and appropriate immunohistochemical stains are used. We conducted a retrospective study of 12 cases of SPNP in our patient population.

## Materials and methods

A retrospective search and analysis of retrieved cases were performed on patients with pancreatic neoplasms referred to or excised at two local tertiary healthcare centers, namely, King Faisal Hospital and Research Center and King Abdulaziz University Hospital in Jeddah, Kingdom of Saudi Arabia, from 2001 to 2021. We identified 12 cases of SPNP that were surgically treated. Two pathologists reviewed the slides, and immunohistochemistry analysis was performed on archived materials; the immunoprofile included antichymotrypsin (A-1-antichymotrypsin rabbit polyclonal antibody, Cell Marque, USA), beta-catenin (β-catenin 14 mouse monoclonal antibody, Cell Marque), CD10 (anti-CD10 (SP67) rabbit monoclonal primary antibody, Ventana, USA), CD56 (anti-CD56 (123C3) mouse monoclonal primary antibody, Ventana), E-cadherin (anti-E-cadherin 36 mouse monoclonal primary antibody, Ventana), ER (anti-estrogen receptor (SP1) rabbit monoclonal primary antibody, Ventana), PR (anti-progesterone receptor (1E2) rabbit monoclonal primary antibody, Ventana), anti-pan keratin (AE1/AE3/PCK26) primary antibody, Ventana), synaptophysin (anti-synaptophysin (SP11) rabbit monoclonal primary antibody, Ventana), NSE ((MRQ-55) mouse monoclonal antibody, Cell Marque), and chromogranin (anti-chromogranin A (LK2H10) primary antibody, Ventana). All immunohistochemical markers were classified as positive or negative. Staining pattern classified as (no staining: 0-1%, focal: 2%-50%, or diffuse: 51%-100%). The clinicopathological data for cases 8-12 (Table 1) have been reported before [4].

**Table 1:** Summary of the clinical findings of the cases of SPNP NET: neuroendocrine tumor, SPNP: solid pseudopapillary neoplasm of the pancreas

Case	Age/sex	Clinical presentation	Location in pancreas	Evidence of metastasis	Follow-up (months)	Note
1	15/Female	Abdominal mass	Tail	No	12	
2	30/Female	Abdominal mass	Tail	Yes (7 years after diagnosis)	84	Initially diagnosed as NET
3	23/Female	Abdominal pain	Head	No	24	
4	15/Female	Abdominal pain and weight loss	Head	No	72	
5	18/Female	Abdominal pain and weight loss	Body	No	36	Initially diagnosed as NET
6	28/Female	Abdominal pain	Tail	No	12	
7	19/Female	Abdominal pain	Head	No	12	
8	19/Female	Abdominal pain	Body	No	143	
9	23/Female	Abdominal pain	Body	No	48	
10	21/Female	Abdominal mass	Tail	No	86	
11	18 /Female	Abdominal pain	Tail	No	24	
12	22/Female	Abdominal mass	Body	No	62	

They were included in this series with additional immunohistochemistry markers and up-to-date clinical follow-up data. The procedures followed were in accordance with the Declaration of Helsinki of 1975, as revised in 2000. As routine practice at both institutions, informed written consent was obtained from each patient to obtain permission to utilize their pathological tissue specimens for laboratory studies. The study was approved by our institution's Research Committee of the Biomedical Ethics Unit (reference no. 455-21).

## Results

We analyzed 12 cases of SPNPs. The summary of the clinical findings of the cases is demonstrated in Table 1. All patients were premenopausal women with an age range from 18-30 years old. Eight patients presented with abdominal pain/discomfort, two of which exhibited weight loss, and four presented with an abdominal mass. The tumor size ranged between 1.5 and 15 cm. All cases were well-circumscribed, and two showed focal areas of hemorrhage. Distinct from our previous study, two referral cases were initially diagnosed as NETs at outside hospitals; one case demonstrates multifocal disease (two separate foci of SPNP). Two referral cases were initially diagnosed as NETs at outside hospitals; one case demonstrates multifocal disease (two separate foci of SPNP). All the cases exhibited a typical microscopic appearance of SPNP. They were composed of sheets of medium-sized, relatively uniform round to oval cells, clear to eosinophilic cytoplasm, and central ovoid nuclei with occasional nuclear grooves. There were no aggressive morphological features, such as cellular atypia, mitosis, tumor infiltration, or vascular and perineural invasion, in any of the cases.

**Figure 1:**
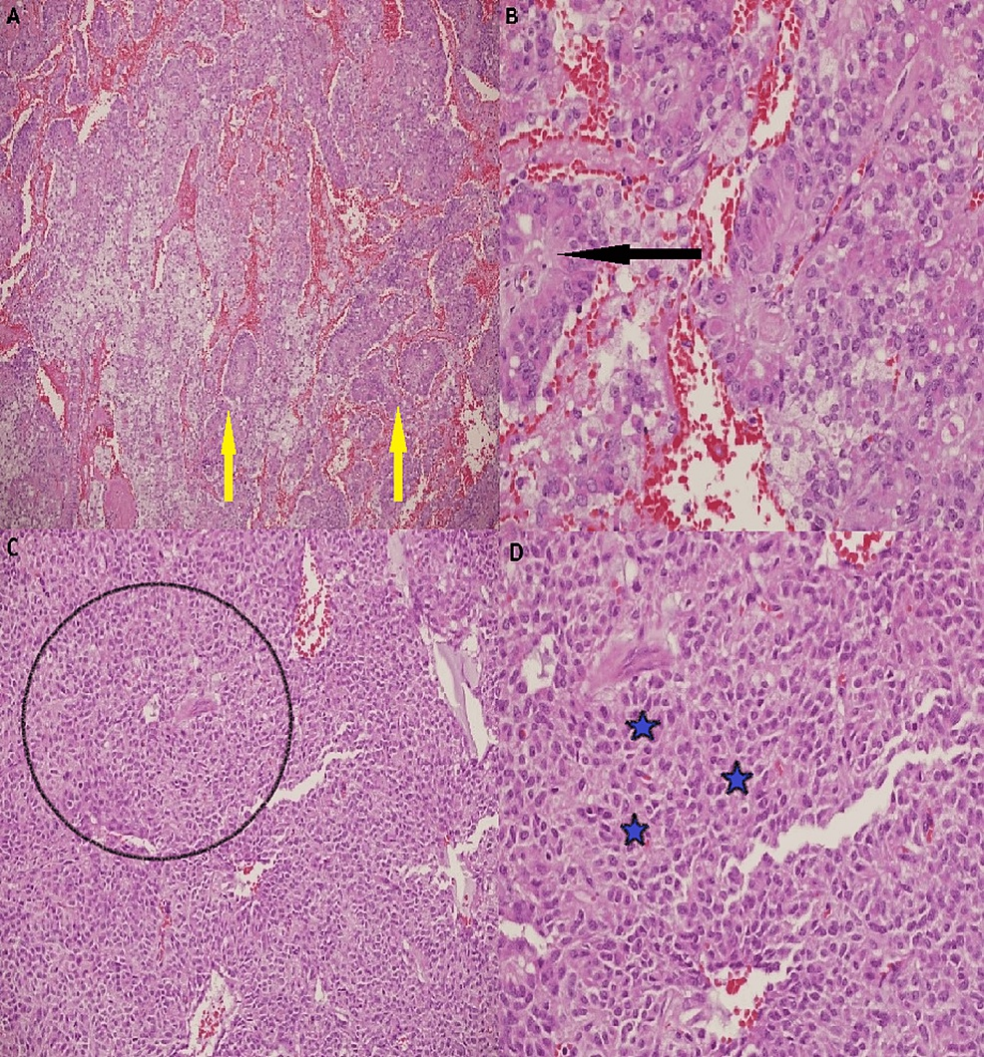
Hematoxylin and eosin-stained sections A. The tumor is comprised of discohesive cells forming a cuff surrounding blood vessels, resulting in a pseudopapillary architecture (yellow arrows) (100). B. Higher power, areas with pseudopapillary architecture (black arrow) (200). C. Tumor areas with a solid pattern (circled area) (100). D. Higher power view, neuroendocrine features with eosinophilic cytoplasm, and central ovoid nuclei (blue asterisk) (200).

**Figure 2:**
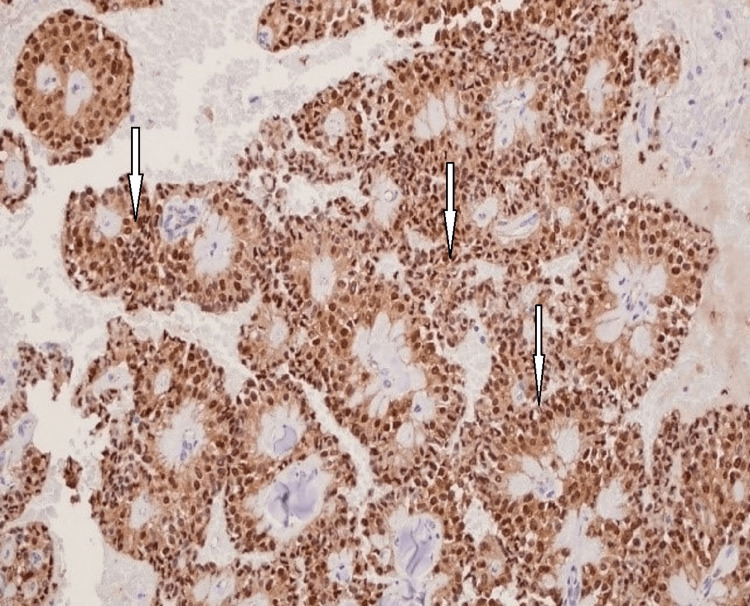
Immunohistochemistry stain for beta-catenin Strong and diffuse positive immunoreactivity (arrows) (10X).

All the cases showed pseudopapillary structures covered by poorly cohesive neoplastic epithelial cells and a few solid areas (Fig. 1A-1D). Two cases demonstrated prominent areas of hemorrhage with foam cells, giant cells, and cholesterol clefts. Immunohistochemistry showed that all 12 cases were positive for vimentin, CD56, CD10, alpha-1-antichymotrypsin, NSE, and β-catenin (Fig. 2). For the majority of the time span of this study, β-catenin was considered the most specific immunohistochemistry stain to confirm the diagnosis of this tumor type.

**Figure 3:**
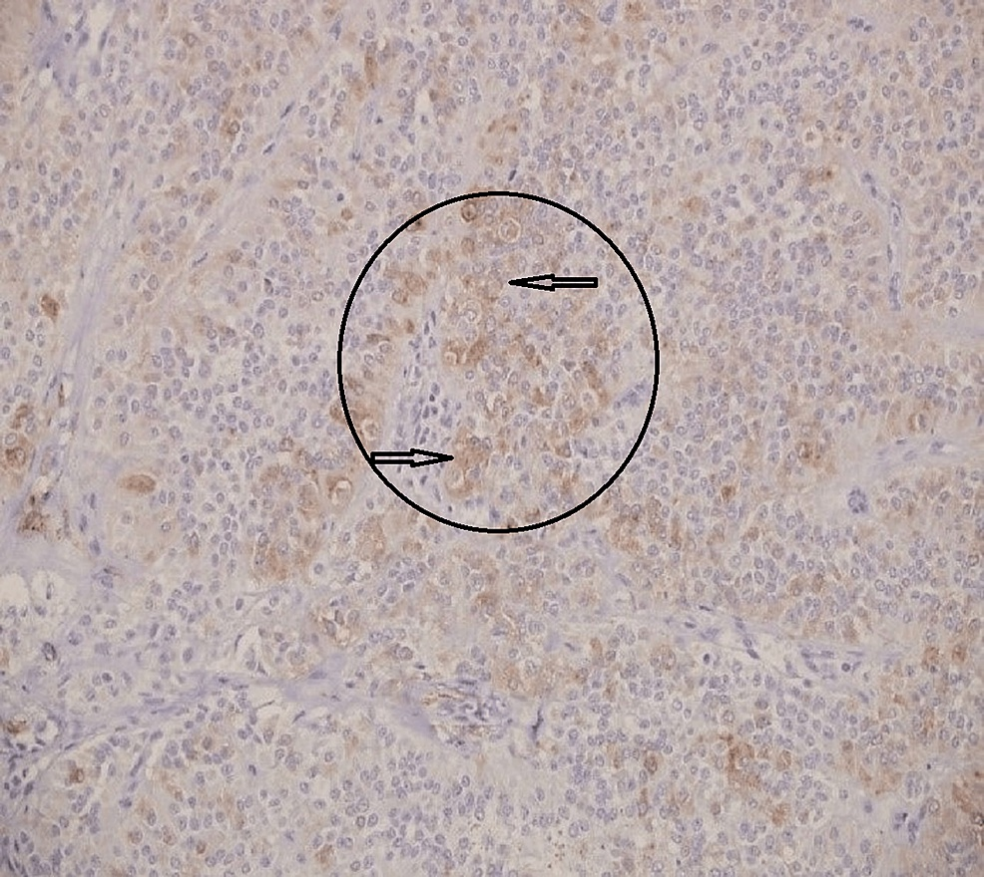
Immunohistochemistry stain for synaptophysin Tumor cells demonstrate a focal positive staining (arrows within the circled area) (10X).

Nine of the cases (9/12) showed positive staining progesterone receptors. Four (4/12) were focally positive for synaptophysin (Fig. 3).

**Figure 4:**
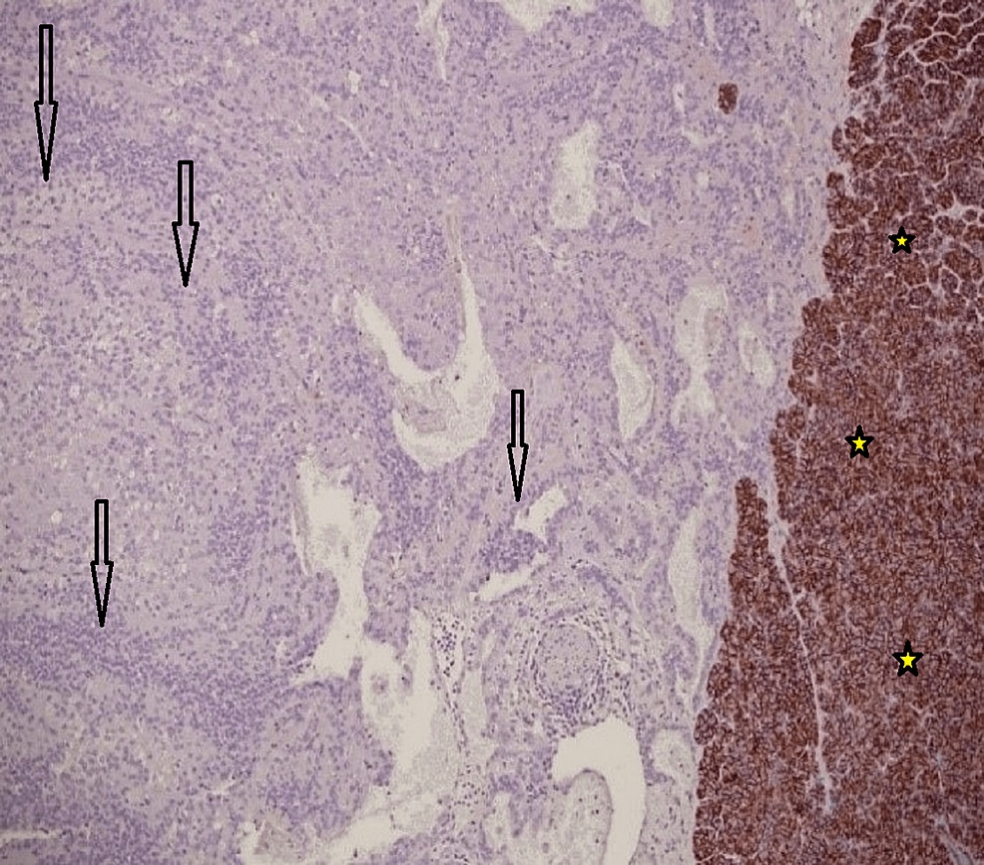
Immunohistochemistry stain for E-cadherin Immunohistochemistry stain for E-cadherin shows negative staining of the tumor cells (arrows) and positive in the non-neoplastic tissue (yellow asterisks) (10X).

All cases were negative for pancytokeratin, estrogen receptors, and chromogranin-A, with aberrant loss of membraneous staining for E-cadherin (Fig. 4). One patient also had an associated ovarian mature cystic teratoma. All patients were treated surgically. Only one patient presented with peritoneal metastasis after seven years of follow-up, but generally, all are doing well.

## Discussion

SPNP is a rare neoplasm with low malignant potential and indolent biological behavior [5-7]. SPNP was first described by Virginia K. Frantz in 1959. Since then, various other names have been used for this type of tumor, including Frantz’s tumor, solid and papillary epithelial neoplasm, solid-cystic tumors, papillary-cystic tumors, and solid pseudopapillary neoplasm of the pancreas. The latter is the most recent terminology used (WHO, 2019). This current study shows that all 12 patients were women aged between 18 and 30 years. Eight patients presented with abdominal pain/discomfort, two of which have demonstrated significant weight loss, and four patients had an abdominal mass. 

Two patients were referred with an initial diagnosis of a well-differentiated NET of the pancreas from outside hospitals. SPNPs were reported in the literature as case reports and a few large series [2,8,9]. More than 90% of the patients were young women. In previous studies, follow-up data indicated that this tumor is a low-grade malignant neoplasm and found primarily in young women [10].

The clinical presentation of SPNP is relatively non-specific. The tumors are usually found incidentally, and they generally cause mild abdominal symptoms, such as abdominal discomfort and acute/chronic pain, and infrequently cause weight loss, nausea, and vomiting. SPNP usually arises in the tail, the body, or occasionally in the head of the pancreas. Rarely, these tumors might rupture and present with hemoperitoneum [11]. Laboratory findings yield little additional diagnostic information and are usually within normal limits. Serum tumor markers (CEA, CA19-9) are usually within normal limits. However, one study observed an elevated serum level of CA19-9 in one out of 18 cases but normal CEA in all of the cases, and two of the study cases were initially diagnosed as NET by cytology [12].

The origin and histogenesis of this tumor are controversial and conjectural [13]. Activation of the Wnt/β-catenin in SPNP is associated with the upregulation of genes required in Notch, Hedgehog, and androgen receptor signaling pathways. The activation of Wnt/β-catenin results in E-cadherin expression changes and glutamate-ammonia ligase (GLUL) expression, indicating its potential as a Wnt target gene [14]. Genetic alterations seen in ductal carcinoma of the pancreas, such as KRAS, TP53, and SMAD4, are not detected in SPNP, indicating that they differ from other pancreatic neoplasms. SPNPs are characterized by the presence of somatic CTNNB1 exon 3 hotspot mutations, leading to stabilization and nuclear localization of β‐catenin, which can be detected by immunohistochemistry. A key component of the Wnt pathway is the protein β‐catenin encoded by the gene CTNNB1, which regulates transcription of downstream target genes (e.g., LEF1 and AXIN2). Hotspot mutations in CTNNB1 can cause an abnormal accumulation of nuclear β‐catenin through stabilization of the protein and eventually constitutive activation of the pathway [15].

In the current series, the tumor size ranged between 1.5 and 15 cm. All the cases were well circumscribed. Hemorrhage and necrosis are common in cystic areas. All the reviewed cases showed the typical microscopic features of SPNP. SPNP histologic appearances are usually distinctive and diagnostic, comprised of solid and pseudopapillary components combined with hemorrhage and pseudocysts changes in variable ratio. The solid tumor element may morphologically resemble NETs, composed of poorly cohesive monomorphic cells with a clear to eosinophilic cytoplasm and central ovoid nuclei with occasional nuclear grooves that adhere to hyalinized or myxoid fibrovascular cores. The pseudopapillary architecture is usually due to the discohesion of tumor cells from the fibrovascular cores. Two cases demonstrated conspicuous areas of hemorrhage with foam cells, giant cells, and cholesterol clefts. No aggressive histological features were seen, such as infiltration into surrounding tissues, no evidence of necrosis, cellular atypia, increased mitotic figures, no vascular invasion, and no perineural invasion in any of the cases.

The immunohistochemistry panel revealed that the tumor cells are positive for vimentin, CD56, CD10, alpha-1-antichymotrypsin, NSE, and β-catenin in all 12 cases. Four cases were focally positive for synaptophysin. The CD56 expression, synaptophysin, and chromogranin have been reported in SPNP and can overlap with predicted expression in pancreatic NETs. Such expression could be diagnostically challenging and a pitfall, resulting in a misdiagnosis, unless interpreted in a broader context.

In both cases, initially diagnosed as an NET at the outside hospitals, the immunohistochemistry panel was minimal and diagnosed mainly based on positivity for CD56 and synaptophysin. 

Earlier studies demonstrated that NETs showed more malignant potential and needed different postoperative treatments than SPNs; therefore, differentiating these two tumors is crucial. The five-year survival rate for patients with SPNP is better than that for patients with NETs (95% of SPNs vs. 65% of NETs) [16,17]. It is not uncommon that SPNP is initially misdiagnosed as NET [18-20]. One study found that among 18 initially diagnosed pancreatic NETs, four have been revised as SPNP [19]. Thus, pathologists must practice extreme caution in interpreting the results of the limited immunohistochemistry panel in SPNP. It is recommended to include other markers, such as β-catenin, E-cadherin, and more recently LFE1, to arrive at the correct diagnosis as they yield high sensitivity and specificity for SPNP [21,22].

The progesterone receptor is usually expressed, and androgen receptors are detected in about 80% of cases; however, estrogen is usually negative [10]. All the study’s cases were positive for β-catenin. In some studies, the expression of markers, such as vimentin, alpha-1-antitrypsin, NSE, and the progesterone receptor, was observed in more than 90% of SPNPs [23]. Some authors reported positive immunostaining of SPNPs for chromogranin A with a dot-like pattern [19]. Our study revealed negative staining for chromogranin in all 12 cases.

The classic immune profile of NETs includes positive staining for synaptophysin, chromogranin A, CD56, NSE, pan-cytokeratin, and E-cadherin, and some cases are positive for vimentin, α-1-antitrypsin, CD10, and β-catenin. Meanwhile, the classic immune profile of SPNPs includes positive staining for vimentin, α-1-antitrypsin, CD10, CD56, NSE, and β-catenin and being negative for pankeratin, chromogranin-A, and E-cadherin. Pathologists should not rely on the expression of CD56 and NSE alone to diagnose NETs, even if also accompanied by focal synaptophysin staining. Other supportive markers are recommended to avoid misdiagnosis of SPNPs as NETs. Based on our results, chromogranin-A, E-cadherin, and β-catenin help confirm the diagnosis of SPNPs and rule out NETs.

Recently, further markers have been reported to help to differentiate these two tumors. Guo et al. reported that all SPNPs are positive for lymphoid enhancer-binding factor 1 (LEF1) and negative for Insulinoma-associated protein 1 (INSM1), while NETs show the opposite results [24]. They suggested that the two markers can accurately distinguish between SPNPs and NETs. Until recently, neither of those markers was routinely available in most diagnostic laboratories, but expanding the utility of LFE1 in hematolymphoid malignancies has made this stain more readily accessible. 

SPNP preoperative diagnosis could be achieved by fine-needle aspiration (FNA) cytology, especially in clinically and radiologically typical examples [25]. SPNPs demonstrate typical CT and MRI features of solid and cystic or solid neoplasms with an irregular density within the capsule, usually sharply circumscribed, well-encapsulated, heterogeneous, and hypodense lesions with areas of high signal intensity corresponding to foci of hemorrhage [3,26,27]. The radiological differential diagnosis of SPNPs includes hemorrhagic pseudocyst, parasitic hydatid cyst, and other common cystic neoplasms of the pancreas, such as serous cystadenoma or cystadenocarcinoma, mucinous cystic neoplasms, and intraductal tubulopapillary neoplasm [28]. However, the distinction between SPNPs and NETs may also represent a diagnostic challenge in cytological FNA evaluation. Awareness of the cytological features and appropriate use of immunohistochemical stains can prevent diagnostic errors [20,29].

One of the patients also had an associated ovarian mature cystic teratoma. All patients were treated surgically. Eleven patients are in good health with a follow-up period between one and 11 years. However, one patient developed peritoneal metastasis after seven years but was otherwise doing well; she was kept under regular follow-up till June 2015, and then she was lost to follow-up until June 2021. Her last CT scan of the abdomen showed multiple omental and peritoneal metastasis. She did not receive any form of systemic therapy. A CT scan-guided biopsy revealed a metastatic neoplasm morphologically and immunophenotypically consistent with SPNP.

Complete resection is the treatment of choice for SPNPs, and the standard therapy should involve a complete removal of the tumor, the associated lymph nodes, the involved pancreas, and any adjacent organs. Local invasion, recurrence, or limited metastases should not be considered contraindications to resection [30,31].

The clinical outcome for SPNPs is usually inert. The long-term follow-up of patients showed that distant metastasis or invasion of the peri-tumoral tissue in and of itself does not indicate aggressive clinical behavior of this tumor [29]. Malignant transformation into undifferentiated carcinoma has been reported to be the only reliable predictor of clinical aggressiveness [32].

Limitations of the study

This is a retrospective study involving a small volume of available cases of solid pseudopapillary neoplasms of the pancreas at our institutions.

## Conclusions

It is crucial for practicing pathologists to be aware that SPNP is an uncommon neoplasm of the pancreas with an indolent clinical behavior. Nevertheless, one must be acquainted with SPNP's unique microscopic features and immunoprofile to distinguish it from other solid or partly cystic pancreatic neoplasms, particularly NETs in young females. Adequate surgical intervention is associated with an excellent prognosis.
